# Lignan from *Alnus japonica* Inhibits Adipocyte Differentiation via Cell Cycle and FOXO1 Regulation

**DOI:** 10.3390/molecules25153346

**Published:** 2020-07-23

**Authors:** Hyejin Lee, Ji Hye Jeong, Jae-Ha Ryu

**Affiliations:** Research Institute of Pharmaceutical Sciences and College of Pharmacy, Sookmyung Women’s University, Seoul 04310, Korea; u9698115@naver.com (H.L.); jjh4415@naver.com (J.H.J.)

**Keywords:** *Alnus japonica*, lignan, 3T3-L1, adipogenesis, FOXO1

## Abstract

In the present study, we isolated a lignan ((−)-(2*R*,3*R*)-1,4-*O*-diferuloylsecoisolariciresinol, DFS) from *Alnus japonica* and evaluated its antiobesity potential in vitro. We also determined its mechanism of action in a mouse pre-adipocyte 3T3-L1 cell line. DFS dose- and day-dependently inhibited adipogenesis by downregulation of adipogenic factors and lipid metabolism-regulating factors during adipocyte differentiation. In particular, DFS suppressed cell cycle-regulating factors and induced G0/G1 cell cycle arrest, implying that it had an inhibitory effect on mitotic clonal expansion which occurred at an early stage of adipogenesis. DFS also suppressed adipogenesis through decreasing Akt phosphorylation and increasing the level of Forkhead box protein-O1 (FOXO1). These results suggest that DFS may be a pharmacological candidate for the development of antiobesity, therapeutic, and nutraceutical products.

## 1. Introduction

Modern life encourages the consumption of excessive calories and, in combination with a sedentary lifestyle, as well as genetic factors, promotes the development of obesity. Obesity creates health risks by inducing other chronic diseases such as diabetic mellitus, hypertension, cardiovascular disease, dyslipidemia, and cancer [1]. There are several antiobesity therapies including bariatric surgery and nonpharmacological and pharmaceutical treatments; however, these all have limitations.

Although antiobesity drugs including orlistat, lorcaserin, phentermine, and topiramate are currently available for weight loss, they occasionally produce side effects, including headache, fatigue, constipation, dysgeusia, insomnia, and hypoglycemia [2]. Therefore, new therapeutic agents that are less toxic and can be used in combination with conventional treatments are urgently needed. This has motivated us to attempt to discover an antiobesity compound from natural products and to define the mechanisms by which it might inhibit fat accumulation.

As adipocytes are cells that specialize in the storage of triglycerides as an energy source, one useful strategy is to identify inhibitors of either fat formation (i.e., adipogenesis) or triglyceride accumulation in adipocytes for the development of antiobesity agents. Diverse natural compounds such as curcumin, berberine, (–)-epigallocatechin gallate ((–)-EGCG), and resveratrol have been reported to counteract obesity by regulating either fat formation or the proliferation of adipocytes [3,4,5,6].

Recently, forkhead box-O1 (FOXO1), one of the FOXO subtypes, has attracted attention as an antiobesity target molecule due to its diverse effects in cell cycle control and adipocyte differentiation [7]. Based on previous studies, FOXO1 was shown to be a tumor suppressor that regulates cell cycles in a variety of human cancers [8,9]. FOXO1 also regulates the cell cycle during adipocyte differentiation [10]. In addition, FOXO1 is most highly expressed in the liver, adipose tissue, and the pancreas as a transcriptional activator. In adipocytes, FOXO1 suppresses adipogenesis by specific and direct binding to the promoter region of peroxisome proliferator-activated receptor subtype-γ (PPARγ), which is an essential transcription factor for adipogenesis. Insulin can augment the phosphorylation of the PI3K/Akt/FOXO1 cascade to induce adipocyte differentiation. Once phosphorylated FOXO1 is separated from the promoter region of PPARγ, it is then followed by exclusion from the nucleus which results in PPARγ transcription. The inhibition of FOXO1 phosphorylation and augmentation of nuclear FOXO1 levels therefore suggests itself as a strategy for the suppression of adipogenesis [7].

*Alnus japonica* (Betulaceae) (AJ) is widely used in Korea, China, and Japan as a medicinal herb and a functional food to treat fever, hemorrhage, diarrhea, and alcoholism. AJ contains abundant diarylheptanoids and lignans that exert diverse pharmacological activities including antioxidant, anti-inflammatory, hepatoprotective, anticancer, and antiobesity activities [11,12]. Sung et al. reported the antiobesity potential of AJ fruit and identified several diarylheptanoids as adipogenesis inhibitors [13]. We previously isolated (−)-(2*R*,3*R*)-1,4-*O*-diferuloylsecoisolariciresinol (DFS) from an AJ stem that showed anticancer activity in a mouse xenograft model of colon cancer. DFS suppressed cancer cell proliferation through downregulating the FoxM1-Wnt/β-catenin pathway [14]. Herein, we report the antiobesity effect of DFS and its regulatory mechanism in an adipocyte culture system.

## 2. Results

### 2.1. A Lignan Isolated from A. Japonica Inhibits Adipocyte Differentiation

**Figure 1:**
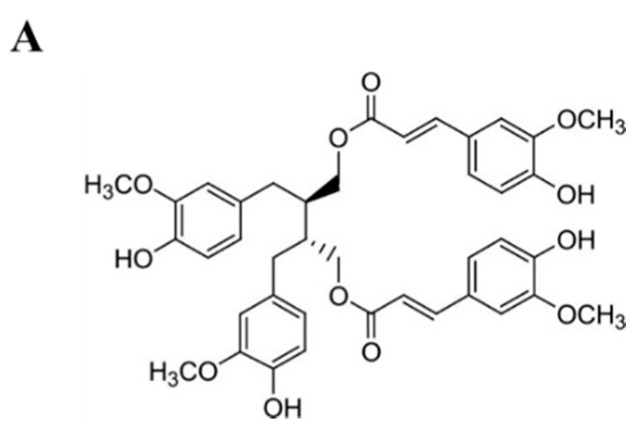

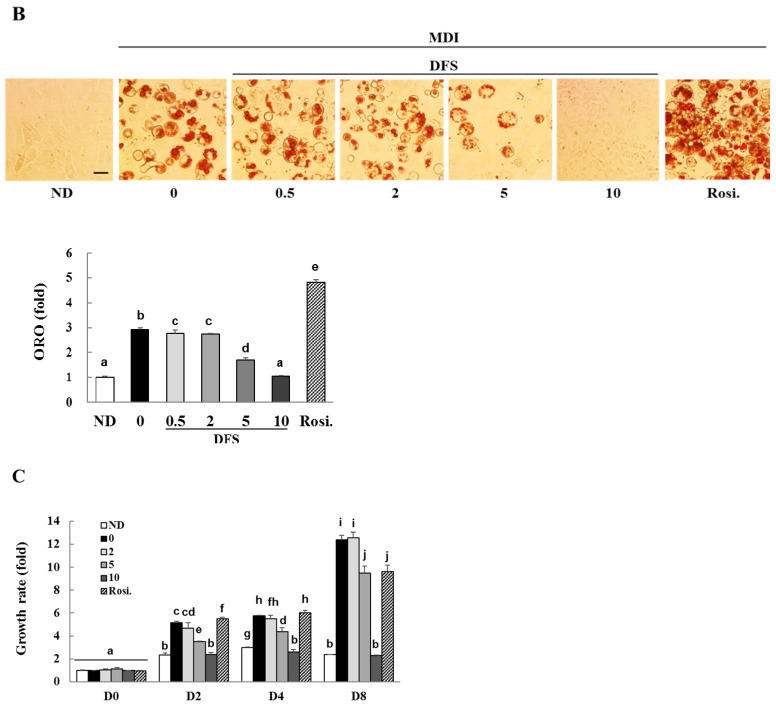
Lignan from *A. japonica* inhibits adipocyte growth and differentiation. (**A**) Structure of lignan ((−)-(2*R*, 3*R*)-1,4-*O*-diferuloylsecoisolariciresinol, DFS). (**B**) 3T3-L1 pre-adipocytes were differentiated with differentiation medium (MDI) in the presence or absence of DFS (0 ~ 10 μM). First, 3T3-L1 cells were stained with Oil Red-O (ORO) on D8 and intracellular lipid contents were quantified and visualized by light microscopy (magnification, ×40). Scale bar = 50 μm. (**C**) MTT assays were performed on differentiation day 0, 2, 4, or 8 (D0, D2, D4, or D8). Rosiglitazone (Rosi., 10 µM) was used as a positive control for adipogenesis. Data are means ± standard deviation (SD) of triplicate experiments. Means without a common superscript differ significantly (*p* < 0.05).

We isolated the lignan (−)-(2*R*,3*R*)-1,4-*O*-diferuloylsecoisolariciresinol (DFS) from a methanol extract of the *Alnus japonica* (AJ) stem; its structure was identified by spectroscopic analysis as previously reported (Figure 1A) [14]. To investigate the antiobesity effect of DFS, mouse preadipocyte 3T3-L1 cells were treated with DFS during adipocyte differentiation. The 3T3-L1 preadipocyte was fully differentiated after chemical stimulation with isobutyl-methylxanthine, dexamethasone, and insulin (MDI), and adipocyte differentiation was evidenced by observation of lipid droplet accumulation [15]. The antiobesity effect of test compounds can, therefore, be determined by measuring lipid formation within adipocytes.

The effects of DFS on lipid formation can be visually quantified using Oil Red-O (ORO) staining on differentiation day 8 (D8). As shown in Figure 1B, the MDI treatment caused intracellular lipid formation, whereas treatment of DFS dose-dependently inhibited lipid formation during differentiation. Adipocytes treated with 10 µM DFS suppressed lipid formation to levels similar to those in cells that were not differentiated (ND). As a positive control, rosiglitazone (10 µM), an agonist of peroxisome proliferator-activated receptor-γ (PPARγ), increased intracellular lipid accumulation compared to MDI [16].

DFS (10 µM) also suppressed adipocyte growth without cytotoxicity during differentiation, as assessed by MTT assay (Figure 1C). As previously reported, MDI treatment increased cell growth compared to the respective ND group on differentiation days 2, 4, and 8 (D2, D4, and D8) [17]. DFS significantly inhibited cell growth by 54.1% (D2), 54.5% (D4), and 81.2% (D8), respectively, when compared to comparable MDI treatment cells. However, the growth rate of DFS-treated cells was not lower than the ND controls on D2, D4 and D8. These results indicate that DFS inhibits differentiation as well as the growth of adipocytes without toxicity.

### 2.2. DFS Suppresses Adipocyte Differentiation via Cell Cycle Arrest during Mitotic Clonal Expansion

**Figure 2:**
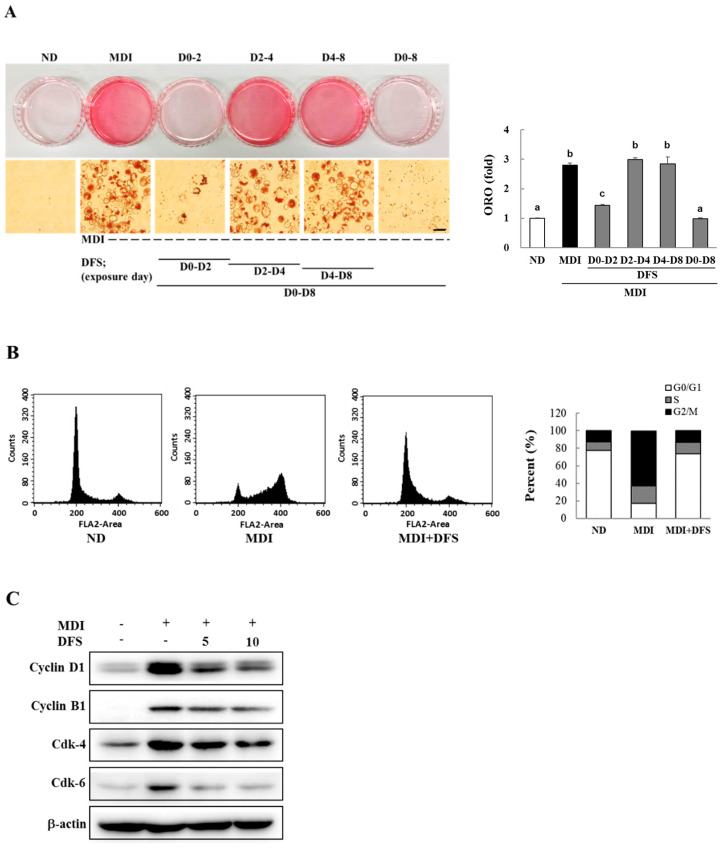
DFS inhibits mitotic clonal expansion during adipocyte differentiation. (**A**) Confluent 3T3-L1 cells were treated with a combination of MDI and DFS (10 μM) and subjected to ORO staining on D8. Scale bar = 50 μm. Data are means ± standard deviation (SD) of triplicate experiments. Means without a common superscript differ significantly (*p* < 0.05). (**B**) Confluent adipocytes were treated with DFS for 48 h followed by flow cytometry. (**C**) Harvested cells on D2 were lysed and subjected to western blotting using indicated antibodies.

Adipocyte differentiation has three stages (early, intermediate, and late stage) [18]; therefore, we investigated which specific stages are affected by DFS. First, 3T3-L1 preadipocytes were treated with 10 µM DFS at different time points during differentiation. These were at D0 ~ D2, D2 ~ D4, D4 ~ D8, and D0 ~ D8. Based on ORO staining, treatment with DFS during D0 ~ D2 completely attenuated lipid formation, while exposure to DFS during the D2 ~ D4 or D4 ~ D8 periods slightly decreased lipid content as compared to MDI (Figure 2A). These data suggest that DFS inhibits differentiation at the early stages.

In general, 3T3-L1 preadipocytes were stimulated by MDI to initiate differentiation upon reaching 100% confluence in culture dishes. During early differentiation (D0 ~ D2), preadipocytes undergo subsequent rounds of cell division referred to as mitotic clonal expansion (MCE) which is accompanied by adipogenic gene expression. The MCE has been reported to be a requisite stage for adipocyte differentiation [19]. Complete adipocyte differentiation is achieved by D8.

To clarify the effect of DFS on MCE, MDI-triggered adipocytes were treated with 10 µM DFS for two days and subjected to flow cytometry analysis. As shown in Figure 2B, MDI alone reduced the proportion of the cell population in the G0/G1 phase (17.7%) compared to the ND group (77.7%). DFS, however, reversed the population of MDI-treated cells in the G0/G1 phase to levels that were similar to ND.

During the MCE stage, adipocytes express cyclins and cyclin-dependent kinase (cdk) at specific times to advance the cell cycle. As the cyclin D1-cdk4/6 complex is essential for the G0/G1-to-S phase transition [20], we measured three proteins following treatment with DFS on D2. DFS (10 µM) inhibited the expression of cyclin D1 (41.4%), cdk-4 (23.9%), and cdk-6 (66.1%) as compared with MDI treatment (Figure 2C). DFS induced G0/G1 cell cycle arrest and downregulated cyclin B1 by 24.9% in cell cycle arrested cells. These results show that DFS inhibits adipocyte differentiation by inducing G0/G1 phase arrest at the MCE stage.

### 2.3. DFS Decreases Gene Expression of Adipogenic Factors during Adipocyte Differentiation

**Figure 3:**
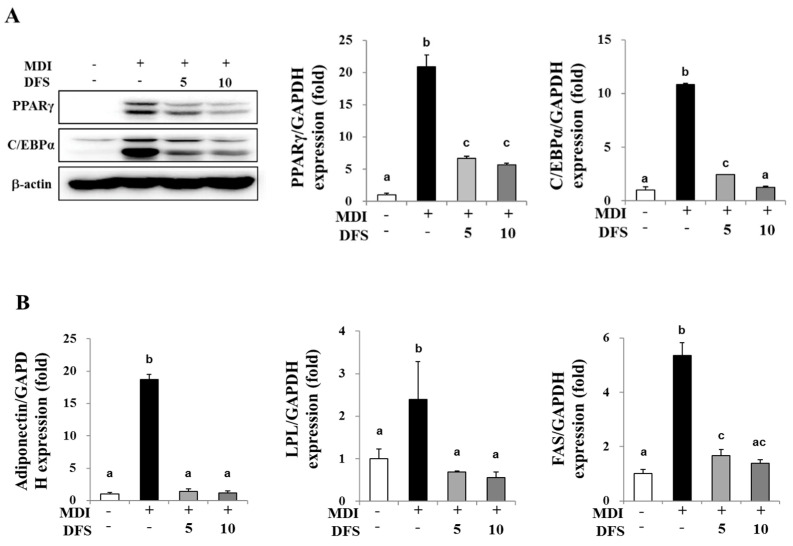
DFS suppresses adipocyte differentiation via modulation of adipogenic gene expression; 3T3-L1 adipocytes were differentiated with MDI containing 5 or 10 µM DFS. (**A**) Cells lysates were prepared on D5 to perform western blotting and quantitative real time PCR (qPCR) to analyze expression levels of peroxisome proliferator activated receptor subtype-γ (PPARγ) and CCAAT/enhancer binding protein-α (C/EBPα). (**B**) The gene expression of adiponectin, lipoprotein lipase (LPL), and fatty acid synthase (FAS) was evaluated by qPCR. Data are means ± standard deviation (SD) of triplicate experiments. Means without a common superscript differ significantly (*p* < 0.05).

The two transcriptional factors, i.e., peroxisome proliferator activated receptor subtype-γ (PPARγ) and CCAAT/enhancer binding protein-α (C/EBPα), are essential for adipogenic gene expression during differentiation [21]. To further examine the inhibitory effect of DFS on adipocyte differentiation, we measured the mRNA or protein levels of PPARγ, C/EBPα, and their target genes by quantitative real time PCR (qPCR) and western blotting analysis. As shown in Figure 3A, DFS decreased protein levels of PPARγ and C/EBPα by 64.8% and 65.4%, and reduced mRNA expression by 73.0% and 88.3%, respectively, as compared to MDI treated cells. After the MCE phase, PPARγ and C/EBPα cooperatively activate several genes involved in adipogenesis. In the present study, DFS reduced the expression of target genes, adiponectin, lipoprotein lipase (LPL), and fatty acid synthase (FAS) by 93.5%, 77.0%, and 74.0%, respectively, as compared to MDI treatment (Figure 3B). Taken together, DFS inhibited adipocyte differentiation via the downregulation of adipogenic factors.

### 2.4. DFS Regulates Akt-FOXO1 Pathway during Adipocyte Differentiation

**Figure 4:**
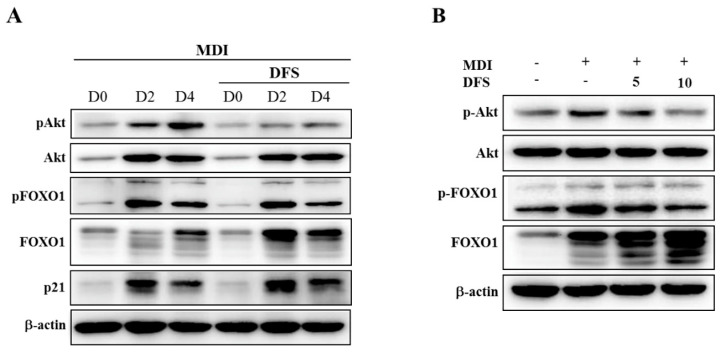
DFS regulates Akt-FOXO1 pathway during adipocyte differentiation. (**A**) Differentiating adipocytes in the presence or absence of DFS (10 μM) were harvested at the indicated periods and analyzed to determine levels of Akt, FOXO, and p21 by western blotting. (**B**) Cells treated with DFS (5 or 10 μM) were harvested on D2 and protein levels of Akt and FOXO1 were analyzed.

Previous studies reported that the Akt-Forkhead box-O1 (FOXO1)-mediated pathway inhibits adipocyte differentiation by downregulating PPARγ [22]. We evaluated the protein levels of Akt and FOXO1 by western blotting analysis to investigate the underlying mechanism of DFS for adipogenic regulation. As DFS regulates the early stage of adipocyte differentiation, the cell lysates were prepared from differentiating cells on D0, D2, and D4. As shown in Figure 4 A, the level of phosphorylated Akt (pAkt) gradually increased in differentiating cells, but this was inhibited by DFS treatment. To assess whether Akt inactivation was required for FOXO1-mediated suppression of adipocyte differentiation by DFS, we observed the expression pattern of FOXO1. The protein level of FOXO1 was increased, and multiple FOXO1 bands appeared as differentiation progressed. DFS treatment further increased FOXO1 level compared to respective day control. Meanwhile, levels of phosphorylated FOXO1 (pFOXO1) were maximized on D2, and slightly reduced in response to DFS treatment as compared to the respective control. Next, we measured the p21 level as an Akt-FOXO1 target gene to clarify the contribution of the Akt-FOXO1 pathway to DFS-mediated inhibition of adipocyte differentiation [23]. FOXO1 has been reported to inhibit adipocyte differentiation by inducing p21 followed by cell cycle arrest in the MCE period [24]. On D2, DFS treatment (10 µM) increased the p21 level in a manner that was consistent with the decreased level of pAkt and pFOXO1 and the increased level of FOXO1.

As shown in Figure 4B, the treatment of DFS dose-dependently inhibited levels of pAkt and pFOXO1 on D2, whereas DFS dramatically increased the FOXO1 level together with the appearance of multiple bands. These data demonstrate that the Akt-FOXO1 pathway contributes to the inhibitory actions of DFS in adipocyte differentiation.

## 3. Discussion

Previous studies demonstrated that mitotic clonal expansion (MCE) is an essential stage of proliferation for adipocyte differentiation [19]. Several factors expressed at the MCE lead the entire differentiation process by inducing the expression of adipogenic genes. Therefore, the modulation of MCE is considered as a strategy for controlling adipogenesis and combating obesity. During MCE, about two rounds of the cell cycle occur, which is initiated by regulatory genes such as cyclins and cyclin-dependent kinases (cdk). At a different phase of the cycle, particular cyclins associate with specific cdks to advance the cell cycle through the checkpoint. Recently, several small molecules were reported to inhibit adipocyte differentiation by preventing further cell cycle progression at the MCE stage [25,26]. For example, isobavachalcone, curcumin, and resveratrol were reported to inhibit cell cycle progression via G0/G1 arrest [4,6,17]. They reduce the expression of cyclin D1, cyclin B1, cdk-4, and cdk-6 in adipocytes, resulting in cell cycle arrest and the suppression of MCE.

Two adipogenic transcriptional factors, the peroxisome proliferator-activated receptor-γ (PPARγ) and the CCAAT/enhancer binding protein-α (C/EBPα), cooperatively express adipogenic genes such as adiponectin, lipoprotein lipase (LPL), and fatty acid synthase (FAS). Consistent with the attenuation of adipocyte differentiation by DFS, the protein and mRNA levels of adipogenic factors were also decreased upon treatment of adipocytes with DFS.

It has previously been reported that FOXO1 is normally expressed as a transcriptional factor in insulin-responsive tissue such as muscle, live, and adipose tissue. In adipocyte differentiation, FOXO1 negatively regulates adipogenesis by binding to the promoter region of PPARγ, resulting in its inactivation. MDI stimulation activates Akt, which subsequently phosphorylates FOXO1 at highly conserved phosphorylation sites (in mice; Thr24, Ser 253, and Ser 316). The phosphorylated FOXO1 (pFOXO1) is designed to be excluded from the nucleus, and then PPARγ transactivation occurs to induce adipogenesis [7]. A well characterized catechin, (–)-EGCG, was reported to inhibit adipocyte differentiation through downregulating the PI3K-Akt-FOXO1 pathway and PPARγ transcription [5]. In this study, DFS reduced the level of pAkt and pFOXO1 to inhibit adipogenesis. In addition, DFS dramatically increased FOXO1 expression together with multiple bands that are responsible for the suppression of adipocyte differentiation.

Consistent with the Akt-FOXO1 regulation, DFS increased p21 expression during adipocyte differentiation. In adipocytes, FOXO1 modulates not only lipogenesis via PPARγ regulation, but also cell cycle progression during adipogenesis. DFS decreased the expression of cyclin D1, cdk-4, and cdk-6, and increased p21 expression, findings that provide a mechanistic explanation for G0/G1 cell cycle arrest by DFS. Further work is needed to uncover the mechanism by which DFS regulates cell cycle-related proteins.

In this study, we isolated a lignan ((−)-(2*R*,3*R*)-1,4-*O*-diferuloylsecoisolariciresinol, DFS) from *Alnus japonica* and assessed its antiobesity potential. DFS suppressed the MCE stage in adipocyte differentiation through inhibiting Akt and FOXO1 phosphorylation and increasing FOXO1 expression.

## 4. Materials and Methods

### 4.1. Isolation of (−)-(2R,3R)-1,4-O-diferuloylsecoisolariciresinol (DFS) from A. Japonica

Air-dried stems of *A. japonica* (5.8 kg) were extracted with methanol and evaporated to dryness. These methanol extracts (294 g) were partitioned with chloroform and water to yield chloroform-soluble fraction. The chloroform-soluble fraction (28 g) was subjected to silica gel column chromatography, followed by reverse phase chromatography to isolate DFS. The purity of DFS was confirmed by high performance liquid chromatography and observation of the ^1^H-nuclear magnetic resonance spectrum [14].

### 4.2. Culture and Pre-Adipocyte Differentiation

The 3T3-L1 preadipocytes used in this study were obtained from American Type Culture Collection (Manassas, VA, USA) and cultured in a DMEM growth medium (WelGENE, Daegu, Korea) supplemented with 10% newborn calf serum (Gibco BRL Life Technology, Grand Island, NY, USA). After cells reached 100% confluence, the growth medium was replaced with a differentiation medium containing MDI (isobutyl-methylxanthine 1 µg/mL, dexamethasone 1 µM, and insulin 1 µg/mL) in DMEM. Two days later, MDI media were replaced with new DMEM containing insulin. After two days of incubation, cells were differentiated in DMEM with 10% fetal bovine serum (FBS, Lonza, Walkersville, MD, USA) and cultured for an additional four days (differentiation day 8).

### 4.3. MTT Assay and Oil Red-O (ORO) Staining

The 3T3-L1 cells were plated into 24-well plates and differentiated in the presence or absence of DFS. On differentiation days 0, 2, 4, and 8, cells were subjected to a 3-(4,5-dimethylthiazol-2-yl)-2,5- diphenyltetrazolium bromide (MTT) (Sigma, St. Louis, MO, USA) assay.

The intracellular lipid content in adipocytes was measured by Oil Red-O (ORO) staining. Cells were fixed and stained with a 0.5% ORO solution, and extracted with 4% Nonidet P-40 to quantify the lipid content. The optical density of the extract was measured at 520 nm on a microplate reader. Images of accumulated lipid drops in mature adipocytes were obtained using an inverted phase-contrast microscope (TH4, Olympus, Tokyo, Japan).

### 4.4. Flow Cytometry

In the presence or absence of DFS, 100% confluent 3T3-L1 adipocytes were incubated with MDI for 48 h and subjected to flow cytometry after staining with propidium iodide (Sigma, St. Louis, MO, USA). Approximately 10,000 cells per sample were analyzed using a FACS Calibur system (Becton Dickinson, San Jose, CA, USA), and cell cycle progression was analyzed with the CELL Quest program.

### 4.5. RNA Extraction and Quantitative Real-Time PCR (qRCR)

Next, 3T3-L1 differentiated with or without DFS were lysed with TriZol reagent (Molecular Research Center, Cincinnati, OH, USA) at differentiation day 5 to purify total RNA, which was then used for cDNA synthesis (Labopass™ cDNA synthesis kit, Cosmogenetech, Seoul, Korea). Then, cDNA was used to evaluate the level of gene expression by qPCR using SYBR Premix Ex TaqTM real time PCR Kit (Cosmogenetech) and Applied Biosystems 7500 Fast Real-Time PCR System (Foster City, CA, USA). Primers used for amplifications are listed in Table 1.

### 4.6. Western Blot Analysis

Cells were harvested and lysed in a buffer (25 mM Tris-Cl, pH 7.5, 100 mM NaCl, 1% NP-40, 1% sodium deoxycholate, 0.1% sodium dodecyl sulfate, and protease inhibitor cocktails) before being centrifuged at 15,000 rpm for 20 min. The total proteins (20 μg) were loaded into a sodium dodecyl sulfate-polyacrylamide gel, electrophoresed, and transferred to PVDF membrane. The membrane was probed with the primary antibody for PPARγ, cyclin D1, cyclin B1, cdk-6, Akt, phospho-Akt, FOXO1, phospho-FOXO1 (Ser 256) (Cell Signaling Technology, Danvers, MA, USA), C/EBPα, p21 (Santa Cruz, Dallas, TX, USA), or cdk-4 (Abcam, Cambridge, UK). The membranes were then incubated with HRP-conjugated antimouse or antirabbit IgG (Santa Cruz) secondary antibodies. The protein levels were visualized with an Enhanced Chemiluminescence detection system (Bio-Rad, Hercules, CA, USA) and quantified using a Fusion Solo system (Vilber Lourmat, Collegien, France). β-actin (Sigma, St. Louis, MO, USA) served as a loading control.

### 4.7. Statistical Analysis

All values are presented as mean ± standard deviation (SD). Differences were assessed using one-way analysis of variance (ANOVA) followed by a Dunnett’s test. All experiments were performed at least three times. Differences with *p* value of less than 0.05 were considered statistically significant.

## Figures and Tables

**Table 1:** Oligonucleotide primer sequences used for the qRT-PCR analysis.

Gene Name	Forward Primer	Reverse Primer
*PPARγ*	AACTCTGGGAGATTCTCCTGTTGA	GAAGTGCTCATAGGCAGTGCAT
*C/EBPα*	TGCACCACCAACTGCTTAG	AAACCATCCTCTGGGTCTCC
*Adiponectin*	TGTAGGATTGTCAGTGGATCTG	GCTCTTCAGTTGTAGTAACGTCATC
*LPL*	AGGACCCCTGAAGACAC	GGCACCCAACTCTCATA
*FAS*	AGCGGCCATTTCCATTGCCC	CCATGCCCAGAGGGTGGTTG
*GAPDH*	TGCACCACCAACTGCTTAG	GGCATGGACTGTGGTCATGAG

PPARγ, peroxisome proliferator activated receptor subtype-γ; C/EBPα, CCAAT/enhancer binding protein-α; LPL, lipoprotein lipase; FAS, fatty acid synthase; GAPDH, glyceraldehyde 3-phosphate dehydrogenase.
